# Clinical and Bacteriological Profile of Neonatal Sepsis: A Prospective Hospital-Based Study

**DOI:** 10.1155/2020/1835945

**Published:** 2020-08-26

**Authors:** Jimba Jatsho, Yoriko Nishizawa, Dorji Pelzom, Ragunath Sharma

**Affiliations:** ^1^Phuentsholing Hospital, Ministry of Health, Bhutan; ^2^Khesar Gyalpo University of Medical Sciences of Bhutan, Thimphu, Bhutan; ^3^Policy and Planning division, Ministry of Health, Thimphu, Bhutan; ^4^Jigme Dorji Wangchuck National Referral Hospital, Thimphu, Bhutan

## Abstract

**Background:**

Neonatal sepsis remains one of the leading causes of mortality and morbidity in developing countries. With a dearth of data on neonatal sepsis in our country, this study was conducted to determine the incidence of clinical neonatal sepsis and evaluate the clinical, bacteriological, and antimicrobial susceptibility profile of organisms. *Material and Methods*. A prospective cross-sectional study was conducted in the Neonatal Unit of the National Hospital from 1^st^ January to 31^st^ December 2016. All neonates admitted with suspected clinical sepsis were included. Sepsis screens and cultures were sent under aseptic conditions. Data was analyzed using STATA™ version 12. Clinical features and neonatal and maternal risk factors were analyzed using chi-squared test. Bacteriological profile was analyzed with descriptive statistics.

**Results:**

During the study period, incidence of culture positive neonatal sepsis was 19 per 1000 admissions with a blood culture positivity rate of 14%. 54.5% had culture-positive early-onset sepsis (EOS). Prematurity (*p* = 0.012), APGAR < 6 (*p* = 0.018), low birth weight (*p* < 0.001), and maternal intrapartum antibiotics (*p* = 0.031) significantly increased risk for culture-positive EOS. Prematurity (*p* < 0.001), low birth weight (*p* = 0.001), and parental nutrition (*p* = 0.007) were significantly associated with increased risk of culture-positive late-onset sepsis. A positive screen had sensitivity of 81.8% and negative predictive value of 87.7%. Gram-negative organisms were most commonly isolated (64.6%). Coagulase-negative *Staphylococci* (31%) were the commonest isolate followed by *Klebsiella pneumoniae* (27%) and *Acinetobacter* (18.8%). Ninety percent of *Acinetobacter* were carbapenem resistant. Gram-negative sepsis had mortality of 88.9%.

**Conclusion:**

Preterm, low birth weight, low APGAR scores, intrapartum antibiotics, and parental nutrition were significantly associated with neonatal sepsis. Coagulase-negative *Staphylococci*, *Klebsiella pneumoniae*, and *Acinetobacter* were the principal causative organisms. Gram-negative organisms had high resistance to commonly used antibiotics.

## 1. Introduction

Neonatal sepsis is defined as a systemic condition of bacterial, viral, or fungal (yeast) origin that is associated with haemodynamic changes and other clinical manifestations and results in substantial morbidity and mortality [1]. The clinical presentations of neonatal sepsis are nonspecific. This includes symptoms like fever, respiratory distress, lethargy/irritability, convulsions, bulging fontanels, refusal to feed, jaundice, bleeding, abdominal distension, and temperature dysregulation [2]. Early-onset sepsis (EOS) presents within 72 hrs of life, and late-onset sepsis (LOS) presents beyond 72 hours of life [1]. EOS presents where the maternal genital tract is the source of ascending infection. Maternal risk factors like premature rupture of membranes (PROM), chorioamnionitis, peripartum fever, urinary tract infection within 2 weeks prior to delivery and prolonged rupture of membranes > 18 hours, multiple gestations, and caesarean sections are associated with increased risk of EOS. LOS occurs as a result of postnatal nosocomial infections or community-acquired infections. The risk factors associated with LOS are prematurity, prolonged invasive interventions like mechanical ventilation and intravascular catheterization, failure of early enteral feeding with breast milk, long duration of parenteral nutrition, hospitalization, surgery, and underlying respiratory and cardiovascular diseases [3]. The spectrum of microbiological pathogens implicated in neonatal sepsis, in developing countries, differs from those occurring in developed countries, with majority contributed by gram-negative organisms in resource-poor areas [4]. Neonatal sepsis claims over 1.5 million infants' lives each year, the majority in sub-Saharan Africa and Southern Asia [5].

Neonatal sepsis still remains the top three cause for neonatal morbidity and mortality globally as well as in Bhutan. NM constitutes 70% of IMR. The major causes of NM are prematurity (38%), neonatal infection (31%), and congenital malformations (16%) [6]. There are no comprehensive researches done so far to study the status of neonatal sepsis in Bhutan. This study was aimed at determining the incidence and risk factors and evaluating the bacteriological profile of neonatal sepsis at Jigme Dorji Wangchuck National Referral Hospital (JDWNRH), Thimphu, Bhutan.

## 2. Material and Methods

### 2.1. Study Design and Setting

This hospital-based prospective cross-sectional study was conducted from January 1^st^ to December 31^st^ 2016 in the Neonatal Unit of JDWNRH. It is the largest tertiary center for neonatal care in Bhutan.

### 2.2. Sampling Population and Procedure

All neonates suspected of having neonatal sepsis and admitted in the neonatal unit were included. Clinical sepsis was diagnosed based on presence of one or more of clinical features. Clinical features considered were fever (≥38.0°C), hypothermia (≤36.5°C), convulsions, lethargy, poor feeding, respiratory distress, vomiting, bulging fontanels, jaundice, and umbilical pus infections. We excluded neonates whose parents declined to give informed consent.

Fully informed and voluntary signed consents were obtained from the parents or attendants. Information sheets were also provided to parents/guardians with full information about the study and its objectives. Discontinuation criteria were considered when the baby got discharged or when the baby expired during the hospital stay. All investigations and procedures were performed as per the standard routine practices in the ward, and no additional interventions were advised as part of the study.

The components of a sepsis screen included a total leucocyte count of <5000/cumm or >20000/cumm [7], an absolute neutrophil count, I : T ratio of ≥0.2, an erythrocyte sedimentation rate (ESR) > 15 mm, and C − reactive protein (CRP) ≥ 1 mg/L [8]. One point five to three sample blood was drawn and inoculated using Brain Heart Infusion broth contained in aerobic BacT/ALERT® PF (Pediatric Fastidious Antimicrobial Neutralization Media, BioMerieux, Inc. Durham, North Carolina, USA) microbial detection system. It was incubated at 37°C and observed for bacterial growth for 5 days. Culture specimens not showing any growth at the end of 5 days were considered sterile. All positive blood cultures were considered a “gold standard” of diagnosis of neonatal sepsis [9].

Antibiotic susceptibility and resistance testing was carried out as per Clinical and Laboratory Standards Institute guidelines for antimicrobial susceptibility testing document [10] except for CONS. Intermediate susceptibility was taken as resistant.

### 2.3. Operational Definitions

- Clinical sepsis (CS) is defined as neonates who have signs and symptoms of neonatal sepsis with or without risk factors
- Culture-positive/proven sepsis (CPS) means neonates who have clinical sepsis with positive blood culture growths
- A positive sepsis screen is defined as having two positive sepsis screen parameters out of five or in situations where one parameter is unavailable, two positive out of four parameters
- A negative sepsis screen is defined as having negative sepsis screen parameters
- Sepsis screening positivity is defined as when either the first or both sepsis screens done 12 to 24 hours apart are positive
- Sepsis screen negativity is when two sepsis screens, done 12 to 24 hours apart, are both negative

### 2.4. Data Collection and Management

Data was collected with a structured interviewer-administered questionnaire and from mother's obstetric records. The questionnaire was prepared after reviewing several relevant, international, and regional literatures on neonatal sepsis. All data were collected at the time of admission through interviewing all mothers whose neonates were admitted to the neonatal unit. Clinical data were obtained by daily assessment of the neonates. Data collected were securely kept with the principal investigator. Data was cleaned for inconsistencies, and coded data was entered using EpiData version 3.1 with proper checks for quality control. Data was duplicated and securely stored on a backup hard drive.

### 2.5. Statistical Analysis

The data was then exported to STATA/SE version 12, where processing and analysis were conducted. Descriptive analysis of risk factors and clinical features of CS and CPS were compared. Comparative statistical analysis was done between CPS cases and culture-negative cases. Chi-squared test was used to determine the *p* value for risk factors associated with early- and late-onset sepsis. All factors with *p* < 0.05 were considered statistically significant. The microbiological data was analyzed with descriptive statistics.

## 3. Results

### 3.1. Incidence

**Figure 1:**
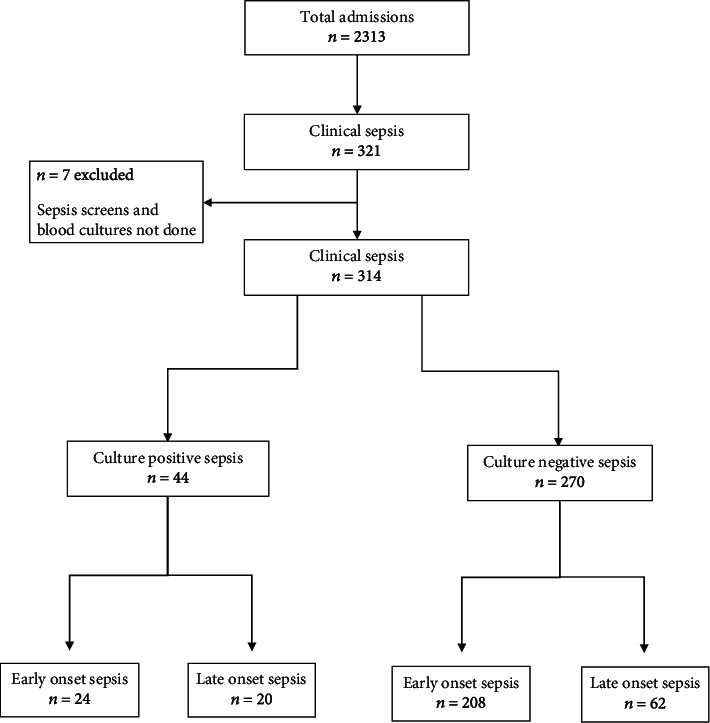
Flow algorithm of neonatal sepsis cases.

In this study, a total of 2313 neonates were admitted to the neonatal unit. Of the total admissions, 321 (13.9%) fulfilled the criteria for clinical sepsis. Majority (66%) were early-onset, culture-negative sepsis (Figure 1).

### 3.2. Baseline Neonatal and Maternal Characteristics

There were 44 culture-positive cases. Majority (52.3%) were males, and male to female ratio was 1.1 : 1 (Table 1).

Majority of mothers whose babies had confirmed CPS had up to 8 antenatal visits (81.8%) and had received intra-partum antibiotics (65.9%) (Table 2).

### 3.3. Clinical Features

Neonates commonly presented with respiratory distress, fever, feeding intolerance, and jaundice. Seizures (*p* < 0.001), respiratory distress (*p* = 0.005), bulging fontanels (*p* = 0.008), hypothermia (*p* = 0.036), and neonatal jaundice (*p* = 0.042) were found to be the significantly associated clinical features for CPS when compared with culture-negative sepsis (see Supplementary Table 1, Additional File 1).

### 3.4. Risk Factors Associated with Neonatal Sepsis

Factors like prematurity (*p* = 0.012), low birth weight (*p* ≤ 0.001), low APGAR scores at 1 (*p* = 0.018) and 5 minutes (*p* = 0.032), and maternal intrapartum antibiotic use (*p* = 0.031) were statistically associated with increased risk of culture-positive EOS (see Supplementary Table 2, Additional File 2). Majority (54.2%) of the EOS culture-positive babies were of low birth weight as compared to EOS culture-negative babies (19.2%), and the difference was highly significant (*p* < 0.001).

Gestational age, birth weight, and use of total parental nutrition (TPN) were statistically associated with late-onset CPS. Prematurity (*p* < 0.001), low birth weight (*p* = 0.001), and administration of TPN (*p* = 0.007) increase the risk for culture-positive LOS (see Supplementary Table 3, Additional File 3).

### 3.5. Validity of Sepsis Screens

Of the 314 sepsis screens, 94% (*n* = 296) screens were complete. A positive septic screen had a sensitivity of 81.8%, specificity of 22.5%, positive predictive value of 15.6%, and negative predictive value of 87.7% with blood culture being considered the gold standard to detect neonatal sepsis (see Supplementary Table 4, Additional File 4).

### 3.6. Microbiological Findings

Blood cultures were done for 314 neonates. 48 organisms were isolated from 44 culture-positive neonates. EOS and LOS had 10.30% (24/232) and 24.4% (20/82) blood culture positivity, respectively. The overall blood culture positivity rate in this study was 14%. Overall, gram-negative organisms were isolated more frequently than gram positives (64.6%). A near equal number of organisms were isolated in EOS (52.1%) and LOS (47.9%).

Blood culture positivity rate for outborn neonates (15.8%) was higher compared to that for inborn neonates. Majority of the organisms (*N* = 40) were isolated from inborn cases (see Supplementary Table 5, Additional File 5). None of the cerebrospinal fluid cultures isolated any organisms.

### 3.7. Antibiotic Susceptibility

Antibiotic sensitivity testing was not done for CONS as it has been routinely considered a contaminant. More than 90% of the *Klebsiella pneumoniae* isolates were found to be resistant to third-generation cephalosporins but carbapenem sensitive. Around 89% of *Acinetobacter* were carbapenem-resistant Acinetobacter (CRAB) (Table 3).

### 3.8. Mortality

There were 26 (8.1%) deaths in the overall cohort of 321. Although around two-thirds (65.4%) of the deaths were culture-negative neonates, the mortality rate among CPS neonates was higher (9/44 = 20.5%) as compared to that among culture-negative sepsis (17/270 = 6.3%). Culture-proven LOS (8/20 = 40%) had higher mortality than-culture proven EOS (1/24 = 4.2%). Out of 26 deaths, 8 deaths were due to gram-negative sepsis which was 30.8% of all mortality and 88.9% of all CPS deaths. *Klebsiella pneumoniae* had higher mortality rate (44.4%) than *Acinetobacter* (33.3%) among CPS.

## 4. Discussions

Our incidence of culture-positive neonatal sepsis was found to be 19 per 1000 neonatal admissions or 1.90% of total admissions. In a recent study from India, a higher incidence of 35.5/1000 admissions was reported [11]. Lu et al. reported a much comparable rate of 10.5 per 1000 admissions [12]. Similar comparative studies are limited as most studies on neonatal sepsis report incidences per 1000 live births. We noted culture-positive EOS (54.5%) was higher than LOS (45.5%). Higher prevalence of EOS was also reported by other studies [13, 14]. Slight male predominance noted was similar to other regional studies [15–17]. This sex difference may be due to a gene located on the X chromosome and involved with the function of the thymus or with synthesis of immunoglobulins in the male infants thus conferring less immunological protection compare to females [18]. Clinically, majority of the neonates presented with respiratory distress, jaundice, and fever similar to other studies [19, 20]. In contrast to this, Chaudhari et al. reported refusal to feed (77.4%) and lethargy (67.9%) as the commonest features in their study [21].

This study showed none of this potential maternal risk factors were found to be statistically associated with CPS except for maternal intrapartum antibiotics (*p* = 0.031). This contradictory results to what is known from international literature [22] may be due to the fact the confirmed EOS in our setting was non-Group B *streptococcus* (GBS). Also, since the number of confirmed EOS in our study is quite small, the true implication of this result may be doubtful.

Neonatal factors like preterm, low birth weight, and low APGAR scores were statistically associated with culture-positive EOS. A recent study in India showed preterms had more CPS [23]. It is known that low birth weight neonates have low IgG levels which make them more prone to infections [24]. Birth weight and gestation are inversely related to sepsis. Similar observations were also noted by other studies [23, 25]. Neonates with low APGAR scores were statistically associated with CPS. Low APGAR scores act as stress factors which make these neonates more prone to infections because of the poor adaptation to extra uterine life.

This study found that neonatal factors such as preterm gestational age, low birth weight, and use of TPN were statistically associated with late-onset CPS. Tsai et al. and Boghossian et al. reported an inverse relationship between incidence of LOS and gestational age [3, 26]. The statistical association of parental nutrition was corroborated by Kung et al. [27].

Our sepsis screens had a high sensitivity (81.8%) which could identify the true sepsis cases early for treatment. However, with low sensitivity, they were not able to detect the true sepsis-negative cases which meant unnecessary use of antibiotics. A high negative predictive value meant that a negative sepsis screen had a high probability of ruling out sepsis. Zaka-ur-Rab et al. showed similar results in his study of sepsis screens [28].

Worldwide records show that the isolation rates on blood cultures vary from 6.7% to 55.4% [29]. Our blood culture positivity rate of 14% was comparable with positivity rates reported by Gupta and Kashyap and Ansari et al. [30, 31]. The low positivity rates could be due to the administration of antibiotics, inadequate or improper sampling, and sepsis due to other causes like fungal, viral, or anaerobic pathogens [32].

Gram-negative organisms were isolated more commonly, corroborating findings by Verma et al., Dalal et al., and Shrestha et al. [23, 33, 34]. In contrast, Galhotra et al. reported gram-positive isolates to be more common [13]. The spectrums of organisms in Southeast Asian countries are different from Western countries where GBS is the predominant pathogen [35]. In line with this trend, our study did not isolate even a single growth of GBS.

*Klebsiella pneumoniae* was the most common cause of gram-negative sepsis followed by *Acinetobacter* spp. similar to other studies [36, 37].

Our study also revealed a large number of organisms exhibiting resistance to many of the antibiotics similar to those also reported in recent studies [38, 39]. We found *Klebsiella pneumoniae* to be 94% resistant to ceftriaxone. On the contrary, Marwah et al. found low cephalosporin resistance which was due to common use of ciprofloxacin and amikacin in its place [40]. Fortunately, all our *Klebsiella i*solates were carbapenem sensitive unlike in a study by Garg et al., where it showed a 100% carbapenem resistance, probably because of the evolving resistance pattern and inappropriate use of high-end antibiotics [41].

Ninety percent of *Acinetobacter* were carbapenem-resistant *Acinetobacter baumannii* (CRAB). However, all were sensitive to polymixin B. The percentage of CRAB has been gradually rising over the last ten years, and outbreaks have been reported worldwide [42]. Similar findings were noted in a study from Pakistan [43]. The high percentage of CRAB in our study is very concerning as serious therapeutic problems arise, as the choice of antibiotics was limited in our setting. Presence of susceptible patients, potentially colonized, selective pressure from antimicrobial use, and poor infection control practices in the NICU may be certain causes [44]. Interestingly, around one-third of these organisms were found in EOS, indicating a possible vertical transmission from mother to child. This was similar to the results by Chakkarapani et al. [45].

In the present study, there were 20.5% deaths among those with CPS which was similar to Kayange et al.'s study [46]. We had higher mortality rate in CPS and in gram-negative septicemia, commonest being *Klebsiella pneumoniae* similar to that noted by Chaudhari et al. [21] in India, further strengthening the results of the present study as the hospital setting maybe comparable.

### 4.1. Limitations

Since there was no normal case control analysis, the risk factor analysis was weak. Improper collection technique, unavailability of neonatal culture microvials, and anaerobic cultures may have limited the blood culture positivity.

## 5. Conclusions

Incidence of neonatal sepsis and blood culture positivity rate was similar to that within the region. EOS was more common than LOS. Preterm, low birth weight, low APGAR scores, and use of intrapartum antibiotics were factors significantly associated with the increased risk of EOS. Preterm, low birth weight, and TPN use were found to increase the risk of LOS. Sepsis screens were highly sensitive to detect true sepsis cases. The high detection of multidrug-resistant organisms and suspected nosocomial infections requires comprehensive and systematic infection control measures. An efficient antibiotic stewardship programme strategy with interdepartmental liaison is needed for routine longitudinal surveillance of antimicrobial susceptibility patterns to guide empirical and rational use of appropriate antibiotics in the intensive care setting.

## Figures and Tables

**Table 1:** Description of baseline neonatal characteristics between clinical sepsis and culture-positive sepsis.

Variables	Clinical sepsis (*n* = 321)		Culture-positive sepsis (*n* = 44)		*p* value
	*n*	%	*n*	%	
Sex					
Male	184	57.3	23	52.3	0.526
Female	137	42.7	21	47.7	
Onset of sepsis					
EOS	238	74.1	24	54.5	0.007
LOS	83	25.9	20	45.5	
Place of delivery					
Inborn	283	88.2	38	86.4	0.731
Out born	38	11.8	6	13.6	
Birth weight					
BW < 2.5 kg	87	27.1	26	59.1	<0.001
BW ≥ 2.5 kg	234	72.9	18	40.9	
Gestational age					
Preterm	71	21.1	23	52.2	<0.001
Term	250	77.9	21	47.7	
Mode of delivery					
NVD	219	68.2	20	45.5	0.011
Assisted	10	3.1	2	4.5	
LSCS	92	28.7	22	50	
APGAR score 1 min					
APGAR ≤ 6	55	17.1	14	31.8	0.019
APGAR > 6	266	82.9	30	68.2	
APGAR score 5 min					
APGAR ≤ 6	19	5.9	6	13.6	0.057
APGAR > 6	302	94.1	38	86.4	
Mechanical ventilation					
No	268	83.5	29	65.9	0.005
Yes	53	16.5	15	34.1	
Parental nutrition					
No	284	88.5	33	75	0.013
Yes	37	11.5	11	25	

**Table 2:** Description of baseline maternal characteristics between clinical sepsis and culture-positive sepsis.

Variables	Clinical sepsis (*n* = 321)		Culture-positive sepsis (*n* = 44)		*p* value
	*n*	%	*n*	%	
Maternal age					
<20 years	11	3.4	2	4.5	0.707
≥20 years	310	96.6	42	95.5	
Education					
Illiterate	64	19.9	13	29.5	0.509
Up to secondary	183	57	23	52.3	
Higher education	62	19.3	7	15.9	
Nonformal education	12	3.7	1	2.3	
PROM ≥ 18 hours					
No	231	72	35	79.5	0.346
Yes	87	27.1	8	18.2	
Unknown	3	0.9	1	2.3	
Antenatal visits					
≥8 visits	297	92.5	36	81.8	0.019
<8 visits	24	7.5	8	18.2	
Foul-smelling liquor					
No	312	97.2	44	100	0.607
Yes	9	2.8	0	0	
Maternal fever					
No	298	92.8	41	93.2	0.933
Yes	23	7.2	3	6.8	
Chorioamnionitis					
No	318	99.1	44	100	1.000
Yes	3	0.9	0	0	
Maternal UTI					
No	315	98.1	44	100	1.000
Yes	6	1.9	0	0	
Multiple PV examination					
≥5 times	103	32.1	9	20.5	0.117
<5 times	218	67.9	35	79.5	
Intrapartum IV antibiotics					
No	170	53	15	34.1	0.019
Yes	151	47	29	65.9	

**Table 3:** Antibiotic susceptibility and resistance profile of isolated organisms.

Antibiotics		*Acinetobacter*	*Klebsiella pneumoniae*	*Escherichia coli*	*Klebsiella oxytoca*	*Citrobacter*	*Streptococcus pneumoniae*	*Enterococcus*
		*n* = 9 (%)	*n* = 13 (%)	*n* = 6 (%)	*n* = 1 (%)	*n* = 2 (%)	*n* = 1 (%)	*n* = 1 (%)
AMP (ampicillin)	S	—	00	1 (16.7)	00	00	—	1 (100)
	R	—	13 (100)	5 (83.3)	1 (100)	2 (100)	—	00
CZO (cefazolin)	S	—	00	1 (16.7)	00	00	—	—
	R	—	13 (100)	5 (83.3)	1 (100)	2 (100)	—	—
CAZ (ceftazidime)	S	1 (11.1)	—	—	—	—	—	—
	R	8 (88.9)	—	—	—	—	—	—
CRO (ceftriaxone)	S	—	1 (7.7)	2 (33.3)	00	1 (50)	—	—
	R	—	12 (92.3)	4 (66.7)	1 (100)	1 (50)	—	—
IPM (imipenem)	S	1 (10)	13 (100)	6 (100)	1 (100)	—	—	—
	R	8 (88.9)	00	0	00	—	—	—
AMK (amikacin)	S	4 (44.4)	10 (76.9)	6 (100)	1 (100)	—	—	—
	R	5 (55.6)	3 (23.1)	00	00	—	—	—
GEN (gentamicin)	S	2 (22.2)	8 (61.5)	5 (83.3)	00	2 (100)	—	—
	R	7 (77.8)	5 (38.5)	1 (16.7)	1 (100)	00	—	—
CIP (ciprofloxacin)	S	2 (22.2)	9 (69.2)	6 (100)	1 (100)	2 (100)	—	—
	R	7 (77.8)	4 (30.8)	0	00	00	—	—
ERY (erythromycin)	S	—	—	—	—	—	1 (100)	—
	R	—	—	—	—	—	00	—
POL (polymyxin)	S	9 (100)	—	—	—	—	—	—
	R	0	—	—	—	—	—	—
PEN (penicillin)	S	—	—	—	—	—	1 (100)	00
	R	—	—	—	—	—	00	1 (100)

S: susceptible; R: resistant.

## Data Availability

The data sets used and/or analyzed during the current study are available from the corresponding author on reasonable request.

## Abbreviations

APGAR: Appearance Pulse Grimace Activity Respiration
CONS: Coagulase negative *Staphylococcus aureus*
CPS: Culture proven/positive sepsis
CS: Clinical sepsis
CRAB: Carbapenem-resistant *Acinetobacter baumannii*
CRP: C-reactive protein
EOS: Early-onset sepsis
ESR: Erythrocyte sedimentation rate
GBS: Group B *Streptococcus*
IMR: Infant mortality rate
JDWNRH: Jigme Dorji Wangchuck National Referral Hospital
LBW: Low birth weight
LOS: Late-onset sepsis
NICU: Neonatal intensive care unit
NM: Neonatal mortality
PROM: Premature rupture of membranes
TPN: Total parental nutrition.

## Supplementary Materials

Supplementary 1 Supplementary Table 1, Additional File 1: statistical analysis of clinical features of neonates with culture-proven sepsis. Seizures, respiratory distress, bulging fontanels, hypothermia, and neonatal jaundice were found to be the significantly associated clinical features for culture-positive sepsis when compared with culture-negative sepsis.

Supplementary 2 Supplementary Table 2, Additional File 2: risk factors associated with neonates with early-onset sepsis. Prematurity (*p* = 0.012), low birth weight (*p* = <0.001), and low APGAR scores at 1 (*p* = 0.018) and 5 minutes (*p* = 0.032) and maternal intrapartum antibiotic use (*p* = 0.031) were statistically associated with increased risk culture positive EOS (*N* = 24).

Supplementary 3 Supplementary Table 3, Additional File 3: risk factors associated with neonates with late-onset sepsis. Prematurity (*p* < 0.001), low birth weight (*p* = 0.001), and administration TPN (*p* = 0.007) were statistically associated with increased risk for culture positive LOS (*N* = 20).

Supplementary 4 Supplementary Table 4, Additional File 4: relationship between septic screens and blood culture. Among 231 positive sepsis screens, 36 had positive blood cultures, and out of 65 negative sepsis screens, 8 were blood culture positive.

Supplementary 5 Supplementary Table 5, Additional File 5: causative organisms of neonatal sepsis. Isolated organisms from early-onset and late-onset sepsis among inborn and outborn neonates admitted from January 1^st^ to December 31^st^, 2016.
