# Supplementary material for: Clinical and Bacteriological Profile of Neonatal Sepsis: A Prospective Hospital-Based Study
Source: Int J Pediatr. 2020 Aug 26;2020:1835945. doi: 10.1155/2020/1835945 (PMC7481930; doi:10.1155/2020/1835945)
Supplement: Supplementary 1 — Supplementary Table 1, Additional File 1: statistical analysis of clinical features of neonates with culture-proven sepsis. Seizures, respiratory distress, bulging fontanels, hypothermia, and neonatal jaundice were found to be the significantly associated clinical features for culture-positive sepsis when compared with culture-negative sepsis. [file 1835945.f1.docx]

**Supplementary Table 1, Additional File 1**: Statistical analysis of clinical features of neonates with culture proven sepsis.

| Clinical Features | | Culture Positive (n=44) | Culture Negative (n=270) | Total  (n=314) | p-value* |
| --- | --- | --- | --- | --- | --- |
|  |  | **n (%)** | **n (%)** | **n (%)** |  |
| Fever | |  |  |  |  |
|  | **No** | 25(56.8) | 152(56.3) | 177(56.4) | 0.948 |
|  | **Yes** | 19(43.2) | 118(43.7) | 137(43.6) |  |
| Hypothermia | |  |  |  |  |
|  | **No** | 37(84.1) | 252(93.3) | 289(92) | 0.036 |
|  | **Yes** | 7(15.9) | 18(6.7) | 25(8) |  |
| Feeding intolerance | |  |  |  |  |
|  | **No** | 27(61.4) | 187(69.3) | 214(68.2) | 0.297 |
|  | **Yes** | 17(38.6) | 83(30.7) | 100(31.9) |  |
| Lethargy | |  |  |  |  |
|  | **No** | 33(75) | 217(80.4) | 250(79.6) | 0.412 |
|  | **Yes** | 11(25) | 53(19.6) | 64(20.4) |  |
| Jaundice | |  |  |  |  |
|  | **No** | 31(70.5) | 146(54.1) | 177(56.4) | 0.042 |
|  | **Yes** | 13(29.6) | 124(45.9) | 137(43.6) |  |
| Skin infections | |  |  |  |  |
|  | **No** | 43(97.7) | 267(98.9) | 310(98.7) | 0.524 |
|  | **Yes** | 1(2.3) | 3(1.1) | 4(1.3) |  |
| Umbilical pus discharge | |  |  |  |  |
|  | **No** | 43(97.7) | 266(98.5) | 309(98.4) | 0.697 |
|  | **Yes** | 1(2.3) | 4(1.5) | 5(1.6) |  |
| Bulging fontanelle | |  |  |  |  |
|  | **No** | 42(95.5) | 269(99.6) | 311(99) | 0.008 |
|  | **Yes** | 2(4.6) | 1(0.4) | 3(1) |  |
| Seizures | |  |  |  |  |
|  | **No** | 37(84.1) | 261(96.7) | 298(94.9) | <0.001 |
|  | **Yes** | 7(15.9) | 9(3.3) | 16(5.1) |  |
| Apnea/Respiratory distress | |  |  |  |  |
|  | **No** | 15(34.1) | 154(57) | 169(53.8) | 0.005 |
|  | **Yes** | 29(65.9) | 116(43) | 145(46.2) |  |

Seizures, respiratory distress, bulging fontanels, hypothermia and neonatal jaundice were found to be the significantly associated clinical features for culture positive sepsis when compared with culture negative sepsis.
