# Supplementary material for: Clinical and Bacteriological Profile of Neonatal Sepsis: A Prospective Hospital-Based Study
Source: Int J Pediatr. 2020 Aug 26;2020:1835945. doi: 10.1155/2020/1835945 (PMC7481930; doi:10.1155/2020/1835945)
Supplement: Supplementary 2 — Supplementary Table 2, Additional File 2: risk factors associated with neonates with early-onset sepsis. Prematurity (p = 0.012), low birth weight (p = <0.001), and low APGAR scores at 1 (p = 0.018) and 5 minutes (p = 0.032) and maternal intrapartum antibiotic use (p = 0.031) were statistically associated with increased risk culture positive EOS (N = 24). [file 1835945.f2.docx]

**Supplementary Table 2, Additional File 2**: Risk factors associated with neonates with early onset sepsis.

| Variables | | | Culture Positive n=24 | | Culture Negative n=208 | | p-value* |
| --- | --- | --- | --- | --- | --- | --- | --- |
|  | | | **n** | **%** | **n** | **%** |  |
| Maternal Age | | |  |  |  |  |  |
|  | **< 20 years** | | 2 | 8.3 | 7 | 3.4 | 0.224 |
|  | **>20 years** | | 22 | 91.7 | 201 | 96.6 |  |
| Maternal Education level | | |  |  |  |  |  |
|  | **Illiterate** | | 5 | 20.8 | 37 | 17.8 | 0.808 |
|  | **Up to Secondary** | | 15 | 62.5 | 122 | 58.7 |  |
|  | **Higher education** | | 3 | 12.5 | 41 | 19.7 |  |
|  | **Non-formal education** | | 1 | 4.2 | 8 | 3.9 |  |
| Maternal PROM > 18 hours | | |  |  |  |  |  |
|  | **Yes** | | 6 | 25.0 | 68 | 32.7 | 0.427 |
|  | **No** | | 18 | 75.0 | 138 | 66.3 |  |
|  | **Unknown** | | 0 | 0 | 2 | 1.0 |  |
| Maternal antenatal visits | | |  |  |  |  |  |
|  | **ANC>8** | | 19 | 79.2 | 163 | 78.4 | 0.928 |
|  | **ANC<8** | | 5 | 20.8 | 45 | 21.6 |  |
| Multiple PV examinations | | |  |  |  |  |  |
|  | **PVE > 5** | | 7 | 29.2 | 75 | 36.1 | 0.504 |
|  | **PVE <5** | | 17 | 70.8 | 133 | 63.9 |  |
| Maternal chorioamnionitis | | |  |  |  |  |  |
|  | **Yes** | | 0 | 0 | 3 | 1.4 | 0.554 |
|  | **No** | | 24 | 100.0 | 205 | 98.6 |  |
| Foul smelling liquor | | |  |  |  |  |  |
|  | **Yes** | | 0 | 0 | 7 | 3.4 | 0.361 |
|  | **No** | | 24 | 100.0 | 201 | 96.6 |  |
| Maternal fever | | |  |  |  |  |  |
|  | **Yes** | | 1 | 4.2 | 17 | 8.2 | 0.487 |
|  | **No** | | 23 | 95.8 | 191 | 91.8 |  |
| Maternal UTI | | |  |  |  |  |  |
|  | **Yes** | | 0 | 0 | 4 | 1.9 | 0.493 |
|  | **No** | | 24 | 100.0 | 204 | 98.1 |  |
| Maternal Intrapartum antibiotics | | |  |  |  |  |  |
|  | **Yes** | | 17 | 70.8 | 99 | 47.6 | 0.031 |
|  | **No** | | 7 | 29.2 | 109 | 52.4 |  |
| Mode of Delivery | | |  |  |  |  |  |
|  | **Vaginal Delivery** | | 14 | 58.3 | 155 | 74.5 | 0.091 |
|  | **Caesarean section** | | 10 | 41.7 | 53 | 25.5 |  |
| Place of Delivery | | |  |  |  |  |  |
|  | | **Inborn** | 23 | 95.8 | 192 | 92.3 | 0.53 |
|  | | **Out born/Referred** | 1 | 4.2 | 16 | 7.7 |  |
| Sex | | |  |  |  |  |  |
|  | | **Male** | 13 | 54.2 | 119 | 57.2 | 0.775 |
|  | | **Female** | 11 | 45.8 | 89 | 42.8 |  |
| APGAR score 1 minute | | |  |  |  |  |  |
|  | **<6** | | 9 | 37.5 | 36 | 17.3 | 0.018 |
|  | **>6** | | 15 | 62.5 | 172 | 82.7 |  |
| APGAR score 5 minute | | |  |  |  |  |  |
|  | **<6** | | 4 | 16.7 | 11 | 5.3 | 0.032 |
|  | **>6** | | 20 | 83.3 | 197 | 94.7 |  |
| Gestational Age | | |  |  |  |  |  |
|  | **Term (> 37 weeks)** | | 13 | 54.2 | 170 | 81.7 | 0.012 |
|  | **Preterm (<37 weeks)** | | 11 | 45.8 | 38 | 18.3 |  |
| Birth Weight | | |  |  |  |  |  |
|  | **Low Birth Weight (<2.5 kgs)** | | 13 | 54.2 | 40 | 19.2 | <0.001 |
|  | **Normal Birth Weight (>2.5 kgs)** | | 11 | 45.8 | 168 | 80.8 |  |

Prematurity (p=0.012), low birth weight (p<0.001) and low APGAR scores at 1 (p=0.018) and 5 minutes (p=0.032) and maternal intra-partum antibiotics use (p=0.031) were statistically associated with increased risk culture positive EOS (N=24).
