# Supplementary material for: Clinical and Bacteriological Profile of Neonatal Sepsis: A Prospective Hospital-Based Study
Source: Int J Pediatr. 2020 Aug 26;2020:1835945. doi: 10.1155/2020/1835945 (PMC7481930; doi:10.1155/2020/1835945)
Supplement: Supplementary 3 — Supplementary Table 3, Additional File 3: risk factors associated with neonates with late-onset sepsis. Prematurity (p < 0.001), low birth weight (p = 0.001), and administration TPN (p = 0.007) were statistically associated with increased risk for culture positive LOS (N = 20). [file 1835945.f3.docx]

**Supplementary Table 3, Additional file 3**: Risk factors associated with neonates with late onset sepsis.

| Variables | Culture Positive n=20 | | Culture Negative n=62 | | p-value* |
| --- | --- | --- | --- | --- | --- |
|  | **n** | **%** | **n** | **%** |  |
| Sex |  |  |  |  |  |
| Male | 10 | 50.0 | 36 | 58.1 | 0.527 |
| Female | 10 | 50.0 | 26 | 41.9 |  |
| Gestational Age |  |  |  |  |  |
| Term | 8 | 40.0 | 54 | 87.1 | <0.001 |
| Preterm | 12 | 60.0 | 8 | 12.9 |  |
| Birth Weight |  |  |  |  |  |
| Normal Birth Weight | 5 | 25 | 45 | 72.6 | 0.001 |
| Low Birth Weight | 15 | 75 | 17 | 27.4 |  |
| Place of Delivery |  |  |  |  |  |
| Inborn | 15 | 72.0 | 46 | 74.2 | 0.943 |
| Out born/Referred | 5 | 25.0 | 16 | 25.8 |  |
| Mechanical Ventilation |  |  |  |  |  |
| Yes | 6 | 30.0 | 12 | 19.4 | 0.317 |
| No | 14 | 70.0 | 50 | 80.6 |  |
| Total Parental Nutrition |  |  |  |  |  |
| Yes | 7 | 35.0 | 6 | 9.7 | 0.007 |
| No | 13 | 65.0 | 56 | 90.3 |  |

Prematurity (p<0.001), low birth weight (p=0.001) and administration TPN (p=0.007) were statistically associated with increased risk for culture positive LOS (N=20).
