# Supplementary material for: Clinical and Bacteriological Profile of Neonatal Sepsis: A Prospective Hospital-Based Study
Source: Int J Pediatr. 2020 Aug 26;2020:1835945. doi: 10.1155/2020/1835945 (PMC7481930; doi:10.1155/2020/1835945)
Supplement: Supplementary 4 — Supplementary Table 4, Additional File 4: relationship between septic screens and blood culture. Among 231 positive sepsis screens, 36 had positive blood cultures, and out of 65 negative sepsis screens, 8 were blood culture positive. [file 1835945.f4.docx]

|  | **Blood CS (+)** | **Blood CS (-)** | Total |
| --- | --- | --- | --- |
| **Septic Screen (+)** | 36 | 195 | 231 |
| **Septic Screen (-)** | 8 | 57 | 65 |
| Total | 44 | 252 | 296 |

**Supplementary Table 4, Additional File 4**: Relationship between septic screens and blood culture.

Among 231 positive sepsis screens, 36 had positive blood cultures and out of 65 negative sepsis screens, 8 were blood culture positive.
