# Supplementary material for: Clinical and Bacteriological Profile of Neonatal Sepsis: A Prospective Hospital-Based Study
Source: Int J Pediatr. 2020 Aug 26;2020:1835945. doi: 10.1155/2020/1835945 (PMC7481930; doi:10.1155/2020/1835945)
Supplement: Supplementary 5 — Supplementary Table 5, Additional File 5: causative organisms of neonatal sepsis. Isolated organisms from early-onset and late-onset sepsis among inborn and outborn neonates admitted from January 1st to December 31st, 2016. [file 1835945.f5.docx]

**Supplementary Table 5, Additional File 5:** Causative organisms of neonatal sepsis.

| Organisms | Inborn | | Outborn | | Total |
| --- | --- | --- | --- | --- | --- |
|  | **EOS** | **LOS** | **EOS** | **LOS** |  |
| *Coagulase-negative staphylococcus* | 7 | 5 | 1 | 2 | 15 (31.3%) |
| *Klebsiella Pneumoniae* | 7 | 4 | - | 2 | 13 (27.1%) |
| *Acinetobacter* | 4 | 4 | - | 1 | 9 (18.8%) |
| *Escherichia Coli* | 4 | 1 | - | 1 | 6 (12.5%) |
| *Citrobacter* | - | 2 | - | - | 2 (4.2%) |
| *Klebsiella Oxytoca* | - | - | - | 1 | 1(2.1%) |
| *Enterococcus* | 1 | - | - | - | 1 (2.1%) |
| *Streptococcus* | 1 |  | - | - | 1 (2.1%) |
| Total Isolates | 24 | 16 | 1 | 7 | 48 |

Causative organisms of neonatal sepsis. Isolated organisms from early onset and late onset sepsis among inborn and outborn neonates admitted from January 1^st^ to December 31^st^, 2016.
